# Niche, Interspecific Association and Community Stability of Understory Vegetation in Artificial Sand-Fixing Forests of the Mu Us Sandy Land

**DOI:** 10.3390/plants15020191

**Published:** 2026-01-07

**Authors:** Huricha Ao, Hongbin Xu, Yuqing Mi, Haibing Wang, Lei Zhang, Shengnan Zhang, Haiyan Gao, Siqi Li

**Affiliations:** 1 College of Desert Control Science and Engineering, Inner Mongolia Agricultural University, Hohhot 010018, China; aohuricha@163.com (H.A.); 13654896836@163.com (S.L.); 2 Key Laboratory of State Forestry and Grassland Administration for Sandy Land Biological Resources Conservation and Cultivation, Inner Mongolia Academy of Forestry Sciences, Hohhot 010010, China; xhb1686@163.com (H.X.); miyuqing2023@163.com (Y.M.); zhangshengnan@163.com (S.Z.); nmghy1993@163.com (H.G.); 3 Key Laboratory of Aeolian Sand Physics and Sand Prevention and Control Engineering of Inner Mongolia Autonomous Region, Hohhot 010018, China

**Keywords:** sand-fixing shrubs, ecological niche, variance ratio, interspecific association, M. Godron stability, vegetation restoration

## Abstract

Understanding the community assembly mechanisms and stability of artificial sand-fixing forests is critical for the management of desert ecosystems. This study investigated the understory vegetation of four artificial sand-fixing shrub forests in the Mu Us Sandy Land to understand community assembly mechanisms and stability by analyzing niche characteristics, interspecific associations, and community stability. The results showed the following: (1) Lc (*Leymus chinensis)*, Ee (*Euphorbia esula)*, Gd (*Grubovia dasyphylla)*, and Ch (*Corispermum hyssopifolium)* all have wide ecological niches and high importance values, serving as key species for maintaining community function. (2) The understory herbaceous plant communities of *S. psammophila*, *A. ordosica* and *C. fruticosum* exhibited low niche overlap, and the *A. fruticosa* understory herbaceous plant community showed high niche overlap. (3) The overall association of the understory herbaceous plant communities of *S. psammophila*, *A. ordosica*, and *C. fruticosum* is positive, while that of the understory herbaceous plant community of *A. fruticosa* is negative; the interspecific associations are weak, and the species show an independent distribution pattern. (4) Among the four understory herbaceous plant communities, the stability of the *S. psammophila* understory herbaceous plant community is relatively the highest, followed by *A. ordosica* and *C. fruticosum* understory herbaceous plant community, and the stability of *A. fruticosa* understory herbaceous plant community is the lowest. Furthermore, community stability was positively correlated with the variance ratio (VR) but negatively correlated with mean niche overlap. We recommend prioritizing *S. psammophila* and *C. fruticosum* for sand fixation and conserving key herbaceous species to optimize resource use and stabilize interspecific relationships. The novelty of this study lies in its integrated assessment of niche characteristics, interspecific associations, and community stability, and it primarily focused on the role of interspecific relationships. Future research should incorporate environmental drivers and shrub functional traits to disentangle the synergistic effects of biotic and abiotic factors, thereby providing a more robust scientific foundation for vegetation restoration in desert ecosystems.

## 1. Introduction

The stability of plant communities is the comprehensive attribute that enables the communities to maintain the stability of structure and function when they are subjected to external disturbances [1]. It is one of the core research directions in current plant ecology [2]. As a key indicator for measuring the anti-interference ability and self-repair potential of an ecosystem, its maintenance mechanism relies on the complex interspecific interactions within the communities. The interspecific relationships within a plant community are mainly manifested through two aspects: ecological niche characteristics and interspecific association [3]. These two factors jointly regulate the stability of the community. Among them, ecological niche breadth represents the breadth of resource utilization by species, while ecological niche overlap degree reveals the similarity in resource utilization among species and the intensity of competition [4]; interspecific association focus on the spatial distribution patterns of species [5], and by analyzing the spatial correlations of species coexistence or exclusion in different habitats, it can directly reflect the impact of habitat differences on species distribution.

The Mu Us Sandy Land, as one of the four major sandy land in China, is located in the transitional zone between arid and semi-arid climates, with scarce water resources, loose surface soil, and a fragile ecological environment [6]. Since the 1978, China has carried out a series of major ecological construction projects, forming large areas of artificial sand-fixing shrub forests, effectively curbing the process of desertification and significantly increasing the vegetation coverage in this region [7,8]. *Salix psammophila* [9,10], *Artemisia ordosica* [11,12], *Corethrodendron fruticosum* [13,14], and *Amorpha fruticosa* [15] are important sand-fixing shrub tree species in the Mu Us Sandy Land, playing an irreplaceable role in desertification prevention [16]. Currently, most studies on the ecological restoration effect of sand-fixing shrubs focus on soil improvement [17,18], water utilization methods [19,20], and soil microorganisms [21,22], while there is a lack of in-depth research on the construction mechanism of plant communities under the protective effect of different sand-fixing shrub forests, the interaction patterns among species, and the overall stability.

To address this research gap, this study takes the understory herbaceous plant communities of four shrub species (*S. psammophila*, *A. ordosica*, *C. fruticosum*, and *A. fruticosa*) in the Mu Us Sandy Land as the research objects. By analyzing the niche characteristics and interspecific association of understory vegetation, and using the M.Godron community stability measurement method, the resource allocation pattern, species coexistence mechanism and stable state of understory plant communities in different shrubs were revealed. This research not only interprets the recovery process and internal laws of the artificial sand-fixing forest ecosystem from the perspective of interspecific relationships, but also provides key scientific support for the scientific selection and reasonable configuration of sand-fixing shrub species in the desertification control of the Mu Us Sandy Land, thereby supporting the sustainable development of the desert ecosystem.

## 2. Results

### 2.1. Analysis of Ecological Niche Characteristics of Plants in Understory of Different Sand-Fixing Shrub Forests

#### 2.1.1. Niche Width

The species ecological niche widths and importance values of the understory herbaceous plant communities in the four shrub forests are shown in Table 1 below. Species were classified as dominant species (DS) or accompanying species (AS) based on their importance value within each community. In the understory herbaceous plant community of *S. psammophila*, Ao is the species with the highest importance value (IV = 19.18%) and a relatively broad niche width (3.60). Lc has the largest niche width (4.07) and a high importance value (IV = 16.34%). Additionally, the dominant species Pt and Ta also exhibit considerable niche widths and importance values, with values of 3.27 (IV = 14.13%) and 2.27 (IV = 13.40%), respectively. Among the accompanying species, Ct has the highest importance value (5.72%) and a relatively broad niche width (2.29); Ip, Cs, Gd, and Sv show moderate importance values and niche widths. The remaining accompanying species, including Aa, Ch, Cc, Ck, Tl, Ac, and Sb, have low importance values and the minimum niche width of 1.00. In the understory herbaceous plant community of *A. ordosica*, Ee is the species with the highest importance value (IV = 32.59%) and the largest niche width (3.12). As dominant species, Pt and Ed have high importance values (25.98% and 20.25%, respectively) and niche widths (2.85 and 2.74, respectively). Among the accompanying species, Sv has a relatively prominent niche width (2.67) but a moderate importance value (7.82%); Gd and Ta have niche widths of 1.80 and 1.47, and importance values of 3.92% and 2.78%, respectively. The remaining accompanying species, including Ct, Cs, Pte, and Sb, have importance values below 5% and the minimum niche width of 1.00. In the understory herbaceous plant community of *C. fruticosum*, Ch is the species with the highest importance value (IV = 32.42%), but its niche width is relatively narrow (1.22). As a dominant species, Gd has the largest niche width (3.58) and a high importance value (21.17%). The accompanying species Sv exhibits a very broad niche width (3.52) with an importance value of 6.68%. Other dominant species include Ao (IV = 20.32%, NW = 2.88) and Aa (IV = 14.12%, NW = 2.00). The remaining accompanying species, including Cc, Ip, and Sc, have low importance values and a niche width of 1.00. In the understory herbaceous plant community of *A. fruticosa*, Ch is the overwhelmingly dominant species, with an importance value as high as 50.50% and a niche width of 2.27. The other two dominant species, Ct and Pa, have importance values of 15.89% and 28.12%, and niche widths of 2.00 and 1.60, respectively. The sole accompanying species, Cf, has an importance value of 5.49% and the minimum niche width of 1.00. At the community level, distinct patterns in niche structure were observed. The understory herbaceous plant community of *S. psammophila* exhibited the widest range of niche breadths alongside a relatively even distribution of importance values among species, indicating a strong overall capacity for resource utilization and pronounced niche differentiation. In contrast, the communities under *A. ordosica* and *C. fruticosum* showed moderate ranges in niche width. The *A. fruticosa* understory herbaceous plant community displayed the narrowest range of niche breadths and the largest disparities in species importance values, suggesting more limited resource utilization capacity and lower functional complementarity within the community.

#### 2.1.2. Niche Overlap

**Figure 1:**
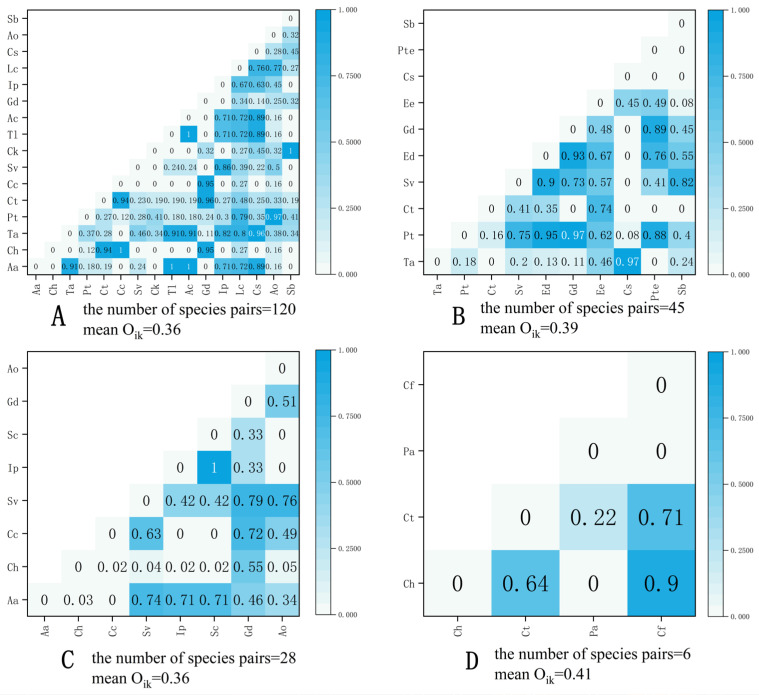
Semi-matrix diagram of species niche overlap of understory herbaceous plant communities under different sand-fixing shrubs. (**A**) stand for the plant community under the *S. psammophila,* (**B**) stand for the plant community under the *A. ordosica,* (**C**) stand for the plant community under the *C. fruticosum,* (**D**) stand for the plant community under the *A. fruticosa*.

The results of niche overlap analysis among species in four different shrub understory herbaceous plant communities are shown in Figure 1. For the plant community under the *S. psammophila* forest (Figure 1A), 120 species pairs among 33 pairs (27.50%) without ecological niche overlap, and there are 55 pairs (45.83%) with O_ik_ < 0.5; there are 32 pairs (26.67%) with O_ik_ > 0.5, the average niche overlap among species in the community was 0.36. For the plant community under the *A. ordosica* forest (Figure 1B), 45 species pairs among 12 pairs (26.67%) without ecological niche overlap, and there are 17 pairs (37.78%) with O_ik_ < 0.5; there are 16 pairs (35.56%) with O_ik_ > 0.5, the average niche overlap among species in the community was 0.39. For the plant community under the *C. fruticosum* forest (Figure 1C), 28 species pairs among 5 pairs (17.86%) without ecological niche overlap, and there are 13 pairs (46.43%) with O_ik_ < 0.5; there are 10 pairs (35.71%) with O_ik_ > 0.5, the average niche overlap among species in the community was 0.36. For the plant community under the *A. fruticosa* forest (Figure 1D), 6 species pairs among 2 pairs (33.33%) without ecological niche overlap, and there is 1 pair (16.67%) with O_ik_ < 0.5; there are 3 pairs (50.00%) with O_ik_ > 0.5, the average niche overlap among species in the community was 0.41. Collectively, these findings suggest that divergent niche structures at the community level among the shrub types could significantly shape the stability and species coexistence dynamics within their respective understory vegetation.

### 2.2. Overall Association of Plants in Understory of Different Sand-Fixing Shrub Forests

The variance ratio method tested the overall interspecific associations in the four understory herbaceous plant communities, as summarized in Table 2. The variance ratios (VR) of the understory herbaceous plant communities of *S. psammophila*, *A. ordosica*, and *C. fruticosum* were 2.67, 2.41, and 1.69, respectively. Among them, the VR statistics of the understory herbaceous plant communities of *S. psammophila* and *A. ordosica* exceeded the χ^2^ critical value range, indicating a significant positive correlation in the interspecific associations; the VR statistics of the understory herbaceous plant community of *C. fruticosum* were all within the χ^2^ critical value range, indicating an insignificant positive correlation in the interspecific associations. The variance ratios of the understory herbaceous plant community of *A. fruticosa* were 0.73, and their VR statistics were all within their respective χ^2^ critical value ranges, indicating an insignificant negative correlation in the interspecific associations. These results suggest contrasting community assembly patterns among shrub forest types.

### 2.3. Interspecific Associations of Plants in Understory of Different Sand-Fixing Shrub Forests

#### 2.3.1. Association Coefficient

**Figure 2:**
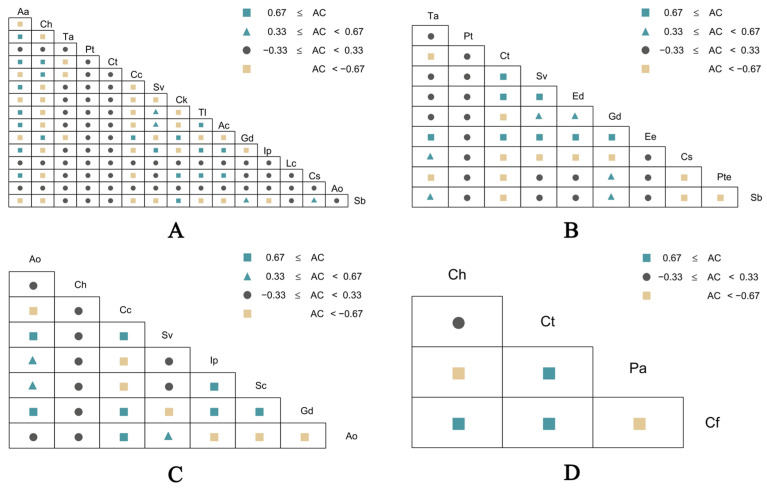
Semi-matrix diagram of species association coefficient (**A**,**C**) of plant communities under different sand-fixation shrubs. (**A**) stand for the plant community under the *S. psammophila*, (**B**) stand for the plant community under the *A. ordosica*, (**C**) stand for the plant community under the *C. fruticosum*, (**D**) stand for the plant community under the *A. fruticosa*.

The interspecific associations within the herbaceous plant communities under the four shrub forests exhibited distinct patterns, as illustrated in Figure 2. For the plant community under the *S. psammophila* forest (Figure 2A), among the 120 species pairs, there are 24 pairs of positive connections, 35 pairs of negative connections, and 61 pairs of no connections, accounting for 20.00%, 29.17%, and 50.83% of the total number of species pairs, respectively. For the plant community under the *A. ordosica* forest (Figure 2B), among the 45 species pairs, there are 14 pairs of positive connections, 12 pairs of negative connections, and 19 pairs of no connections, accounting for 31.11%, 26.67%, and 42.22% of the total number of species pairs, respectively. For the plant community under the *C. fruticosum* forest (Figure 2C), among the 28 species pairs, there are 11 pairs of positive connections, 7 pairs of negative connections, and 10 pairs of no connections, accounting for 39.29%, 25.00%, and 35.71% of the total number of species pairs, respectively. For the plant community under the *A. fruticosa* forest (Figure 2D), among the 6 species pairs, there are 3 pairs of positive connections, 2 pairs of negative connections, and 1 pair of no connections, accounting for 50.00%, 33.33%, and 16.67% of the total number of species pairs, respectively.

#### 2.3.2. Interspecific Correlation

**Figure 3:**
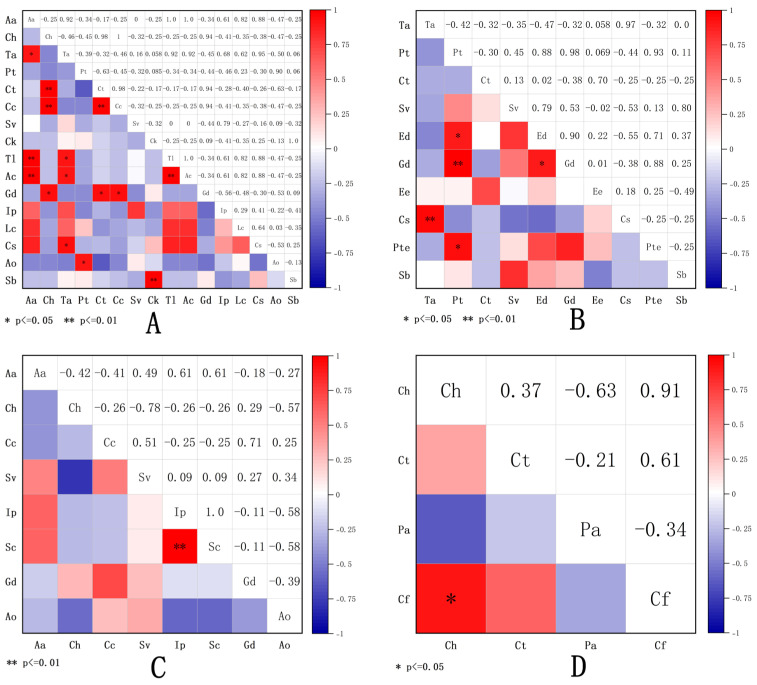
Species correlation coefficient (Pearson) semi-matrix diagram of understory herbaceous plant communities in different sand-fixing shrubs. (**A**) stand for the plant community under the *S. psammophila*, (**B**) stand for the plant community under the *A. ordosica*, (**C**) stand for the plant community under the *C. fruticosum*, (**D**) stand for the plant community under the *A. fruticosa*.

The test results of Pearson correlation coefficient between species in four shrub understory herbaceous plant communities are shown in Figure 3. In the herbaceous plant community under the *S. psammophila* forest (Figure 3A), among the 120 species pairs, 42 pairs were positively correlated (35.00%), 75 pairs were negatively correlated (62.50%), and 3 pairs were not correlated (2.50%). The significant rate of the test was 12.50%. In the herbaceous plant community under the *A. ordosica* forest (Figure 3B), among the 45 species pairs, 24 pairs were positively correlated (53.33%), 20 pairs were negatively correlated (44.44%), and 1 pair was not correlated (6.67%). The significant rate of the test was 11.11%. In the herbaceous plant community under the *C. fruticosum* forest (Figure 3C), among the 28 species pairs, 12 pairs were positively correlated (42.86%), 16 pairs were negatively correlated (57.14%), and there were no uncorrelated pairs.The significant rate of the test was 3.57%. In the herbaceous plant community under the *A. fruticosa* forest (Figure 3B), among the 6 species pairs, 3 pairs were positively correlated (50.00%), 3 pairs were negatively correlated (50.00%), and there were no uncorrelated pairs. The significant rate of the test was 16.67%.

### 2.4. The Stability of Understory Communities in Different Sand-Fixing Shrub Forests and Its Correlation with the Ecological Niche Overlap and Variance Ratio

**Figure 4:**
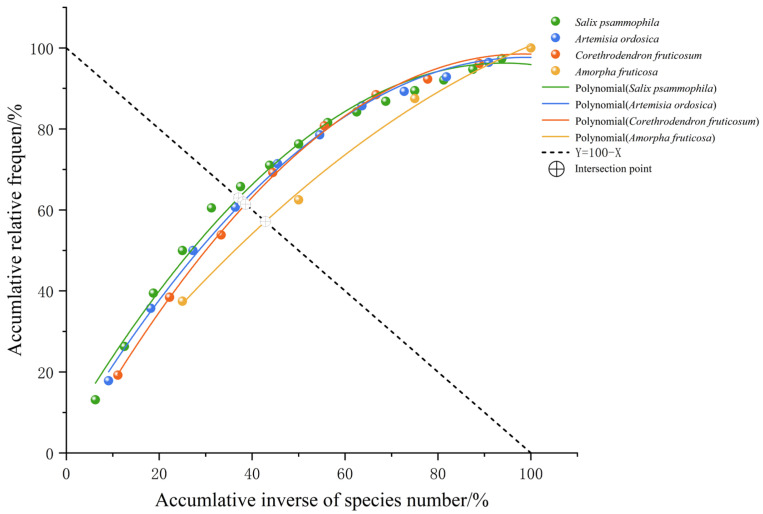
Stability analysis diagram of plant community under different sand-fixation shrubs.

**Figure 5:**
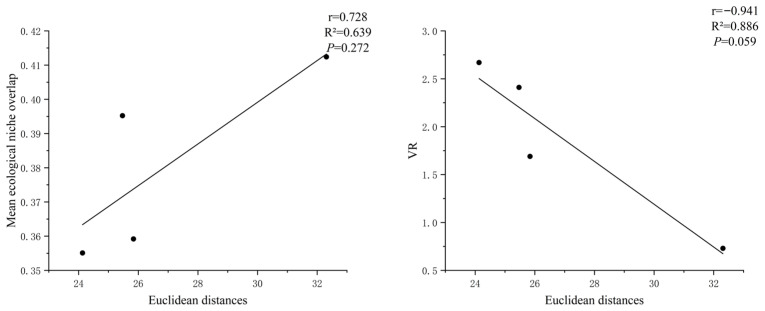
Correlation analysis diagram of the stability of plant communities with the mean ecological niche overlap and the VR.

Using the M. Godron stability assessment method, the stability of four shrub understory herbaceous plant communities was evaluated. The fitted curve equation, determination coefficient (R^2^), intersection coordinates, Euclidean distance from the stable point (20, 80), and test results are shown in Table 3 and Figure 4. The intersection coordinates of the fitted curves of the herbaceous plant communities under the *S. psammophila*, *A. ordosica*, *C. fruticosum*, and *A. fruticosa* undergrowth with the linear equation y = 100 − x are (37.06, 62.94), (38.01, 61.99), (38.27, 61.73), and (42.85, 57.15), respectively. The Euclidean distances from the stable point (20, 80) are 24.13, 25.47, 25.84, and 32.31, respectively. Based on the Euclidean distance, the stability of the plant community under the *S. psammophila* is the highest, followed by the plant communities under the *A. ordosica* and *C. fruticosum*, and the stability of the plant community under the *A. fruticosa* is the lowest. This result is basically consistent with the analysis conclusions of ecological niche characteristics, overall connectivity, and interspecific associations in the previous text. The correlation analysis of the Euclidean distances of four shrub understory plant communities with their average ecological niche overlap and variance ratio is shown in Figure 5. The Euclidean distance is insignificantly positively correlated with the average ecological niche overlap (*p* = 0.272), with a correlation coefficient of 0.728. The Euclidean distance is insignificantly negative correlated with the variance ratio (*p* = 0.059), with a correlation coefficient of −0.941.

## 3. Discussion

### 3.1. Ecological Niche Characteristics of Plant Communities Under Different Sand-Fixing Shrub Forests

This study analyzed the ecological niche characteristics of understory herbaceous plant communities within four artificial sand-fixing shrub forests in the Mu Us Sandy Land. The results are as follows. In the herbaceous plant communities under the four shrub forests, the dominant species occupy relatively high importance values and broad niche widths. The species importance value can indicate the dominance degree of a species in the community, while the ecological niche breadth reflects the species’ ability to utilize resources and its adaptability [23]. In this article, dominant species such as Lc, Ee, Gd, and Ch have a relatively wide ecological niche, indicating that they are widely distributed in sandy areas, have a stronger adaptability to sandy land, and make more efficient use of environmental resources. This might be because these plants can tolerate drought and poor soil conditions. They can absorb water through their well-developed root systems, thereby reducing the impact of water stress on the physiological activities of the plants [24]. Previous research has indicated a proportional relationship between importance value and niche width [25]; however, the findings of this study reveal certain discrepancies from earlier results. Research shows that in the *C. fruticosum* community, the niche width of Ch ranks fifth, while its importance value ranks first. The reason for this result might be that Ch shows an aggregated distribution pattern within the study area.

Ecological niche overlap is used to assess the potential competitive and mutually beneficial relationships between species [26]. Among the four understory herbaceous plant communities, the *S. psammophila*, *A. ordosica*, and *C. fruticosum* understory herbaceous plant communities were predominantly characterized by low niche overlap (O_ik_ < 0.5), accounting for 45.83%, 37.78%, and 46.43% of the total species pairs, respectively. These communities also exhibited low mean niche overlap values of 0.36, 0.39, and 0.36. This indicates that most of the species in these communities currently have a high degree of ecological niche differentiation and are in an independent distribution state. The similarity in resource utilization among species is low, and interspecific competition is not intense. In contrast, the *A. fruticosa* understory herbaceous plant community showed a predominance of high niche overlap (O_ik_ < 0.5), which represented 50% of its species pairs, along with the highest mean niche overlap of 0.41, this indicates that most of the species in this community currently have a relatively low ecological niche differentiation, and the similarity in resource utilization among species is high. Interspecific competition is intense, and the ecological niche overlap is influenced by both the biological characteristics of the species and the habitat. The research found that the ecological niches of understory herbaceous plant communities under *S. psammophila*, *A. ordosica*, and *C. fruticosum* are generally low in overall overlap. On one hand, due to the differences in biological characteristics among different dominant species, which are inevitable due to the varying requirements for light, water, and soil nutrients, there is ecological differentiation, thereby reducing direct competition among them [27]. On the other hand, this might be because the ecological environment of sandy land is fragile, with limited available resources, and the available resources for plants are also limited, resulting in a low degree of ecological niche overlap in the undergrowth plant communities [28].

### 3.2. The Interspecific Associations Characteristics of Plant Communities Under Different Sand-Fixing Shrub Forests

Overall association serves as an important criterion for evaluating both the vegetation recovery process and community stability [29], as it reflects the interactions and connections among species within a community over a specific period [30]. Previous studies have shown that as the process of vegetation restoration progresses, the community will become more stable, the overall association of the community will show a positive connection [31]. This is similar to the results of this study. The overall association of understory herbaceous plant communities under *S. psammophila*, *A. ordosica* and *C. fruticosum* shows a positive connection. Among them, the understory herbaceous plant communities under *S. psammophila* and *A. ordosica* forests have reached a significant level, while the overall connectivity of the understory herbaceous plant community under *A. fruticosa* forest shows a non-significant negative connection.

By analyzing the ratio of positive to negative interspecific associations among species pairs within the understory herbaceous plant communities of different shrubs, we can elucidate the patterns of species coexistence or exclusion [32]. Association coefficient, as a key indicator for measuring interspecific relationships and the stability of community structure, its dynamic changes can to some extent reflect the stable state of the community. Previous studies have shown that as the vegetation restoration process progresses, the interspecific correlations among the dominant species in the community will gradually transition from significant negative correlations to no correlations, and the number of species with no significant correlations will increase. Eventually, in the relatively stable community formed by vegetation restoration, species tend to be distributed independently rather than in a connected manner [33]. This pattern enables species to utilize different non-limiting resources in the habitat relatively independently, achieving rational allocation and efficient utilization of resources, thereby maintaining the stability of interspecific relationships within the community. In this study, among the four understory herbaceous plant communities, the interspecific associations of the species under the *S. psammophila* and *A. ordosica* forests were mainly non-associated, while those under the *C. fruticosum* and *A. fruticosa* forests were mainly positively associated. This indicates that the communities under the *S. psammophila* and *A. ordosica* forests are in a relatively stable state, while those under the *C. fruticosum* and *A. fruticosa* forests are in a relatively unstable state. Combined with the Pearson correlation test, it was found that the overall significant rate of the plant communities under the four shrub forests was extremely low, suggesting that the interspecific relationships were loose and the species within the communities were distributed independently.

### 3.3. The Stability of Plant Communities Under Different Sand-Fixing Shrub Forests the Stability of Plant Communities Under Different Sand-Fixing Shrub Forests and Its Correlation with the Mean Ecological Niche Overlap and Variance Ratio

The findings of HU Er-cha et al. on understory plant communities in different sand-fixing forests of the Mu Us Sandy Land indicate that plant communities formed under the protection of various sand-fixing tree species exhibit significant differences [34,35], which aligns with the results of the present study. However, while previous research on plant communities under the protection of different shrub forests in the Mu Us Sandy Land has mainly focused on post-restoration diversity and community characteristics, this study emphasizes the stability of plant communities under different sand-fixing shrub forests and explains the mechanisms underlying such stability from the perspective of interspecific relationships. The stability assessment revealed a clear gradient among the four understory herbaceous plant communities, with the *S. psammophila* understory community exhibiting the highest stability, followed by community under *A. ordosica* and *C. fruticosum*, while the *A. fruticosa* understory community was the least stable. The marked variation in community stability can be interpreted from both species-specific and community-structural perspectives. At the species level, the strong competitive traits of *A. fruticosa* including high sprouting capacity [36], extensive lateral root development, and superior adaptability, enable it to efficiently sequester water and nutrients. This dominance likely leads to asymmetrical resource allocation within the understory, constraining the growth of subordinate species and thereby undermining overall community stability. In contrast, *S. psammophila* fosters a more favorable microhabitat through its pronounced windbreak and sand-fixing functions [37], coupled with significant litter input [38]. These modifications contribute to a more balanced soil resource environment, which supports higher species coexistence and enhances community-level stability. At the community level, the observed correlation patterns provide further mechanistic insight. Community stability was positively correlated with the mean niche overlap but negatively correlated with the variance ratio (VR). This indicates that more stable communities are characterized by lower overall niche overlap among species and a higher degree of positive interspecific association [39,40]. These patterns align with classic models of ecological succession: during initial restoration phases, colonizing species often exhibit broad, overlapping niches and competitive (negative) interactions. As succession proceeds, niche differentiation increases, direct competition decreases (reducing niche overlap), and interspecific relationships tend to shift toward neutral or positive associations, thereby promoting community stability.

The overall association test results of the four shrub understory plant communities showed that the plant communities under *S. psammophila*, *A. ordosica*, and *C. fruticosum* all exhibited positive connectivity, indicating well-differentiated ecological niches and weak interspecific competition among the species in these understory herbaceous plant communities. However, the stability test results in this study were generally lower. This appears inconsistent with previous research concluding that overall interspecific association tends to show a significant positive correlation when a community becomes stable [39]. The reason may be that the maintenance of community stability depends not only on species composition and community structure but is also affected by external environmental factors [40]. During vegetation restoration in arid regions, the main source of water required for plant growth is atmospheric precipitation [41], making precipitation a key factor determining vegetation stability. The soil in the Mu Us Sandy Land is characterized by high sand content, poor water retention capacity, and intense evaporation of soil moisture [42]. Unstable precipitation further exacerbates the volatility of soil moisture, which may hinder the vegetation from achieving overall community stability.

## 4. Materials and Methods

### 4.1. Overview of the Study Area

**Figure 6:**
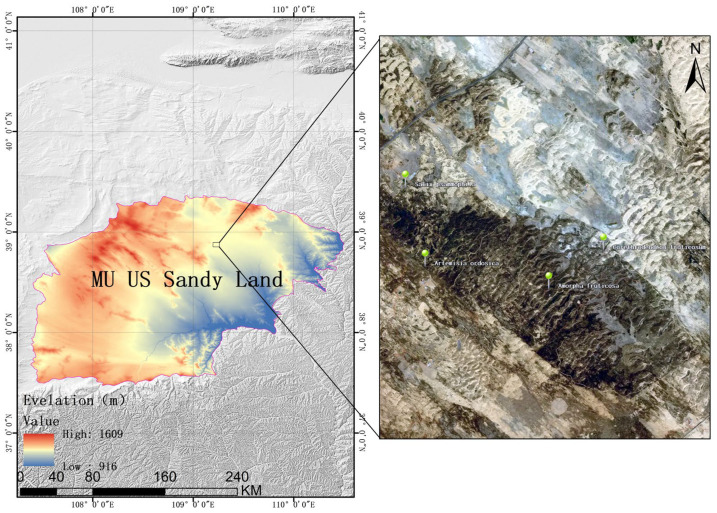
The distribution map of the four shrub forest survey plots.

The study area is located in Wushen Banner, Ordos City, Inner Mongolia Autonomous Region, in the hinterland of the Mu Us Sandy Land (37°38′~39°23′ N, 108°17′~109°40′ E, altitude 1350~1437 m). The distribution map of the four shrub forest survey plots is shown in Figure 6. The study area has a temperate continental monsoon climate [43], the temperature varies greatly between day and night, with an average temperature of 8.6 °C, the annual sunshine duration is 2700 to 3000 h, the effective accumulated temperature is 2800 to 3000 degrees Celsius, and the annual average precipitation is between 350 and 400 mm [44], the annual average wind speed is 4 m/s, the annual evaporation volume ranges from 2200 to 2800 mm, the frost-free period lasts from 113 to 156 days, and the four major climatic characteristics of this region are obvious temperature changes, dry climate, frequent strong winds and intense sunlight [45]. The soil types are mainly sandy soil and calcareous loam. Their characteristics include poor cohesion, loose structure, high permeability, few soil microorganisms and low fertility [46]. The vegetation type in the study area is mainly xerophytic vegetation, in terms of distribution range, herbaceous plants, small shrubs and semi-shrubs are the most widespread, significantly outnumbering trees. The perennial herbaceous and shrub species such as *C. fruticosum*, *A. ordosica*, *S. psammophila*, *Aster altaicus* and *Stipa caucasicaare* considered as dominant and community-forming species in the ecosystem, Occasionally, one can also come across tree species such as the *Salix matsudana* and the *Pinus sylvestris* [47].

### 4.2. Plot Setup and Survey Methodology

In August 2024, the research team conducted a vegetation survey and sampling of the herbaceous plant communities under the artificial shrub forests in the area. First, four shrub plots were selected, namely *S. psammophila*, *A. ordosica*, *C. fruticosum*, and *A. fruticosa*, which were constructed during the period from 2013 to 2015 and had basically the same growth conditions. In each type of shrub forest, a large sample plot of 10 m × 10 m was delineated using a sampling line, and 5 representative 1 m × 1 m herbaceous investigation plots were set within it. There were 5 herbaceous sample plots for each shrub type, and a total of 20 sample plots in the study area. In each 1 m × 1 m herbaceous sample plot, the species of all plants were identified and the individual numbers were counted. At the same time, the natural height of the plants was measured using a tape measure. Then, the plants were cut to the ground level by species, and all plant samples were placed in paper bags. After being brought back to the laboratory, the plant materials were dried in a Laboratory Plant Drying Oven (Grieve Corporation, Round Lake, IL, USA) constant temperature at 65 °C until a constant weight was reached, and the dry weight was determined using analytical balances (±0.01 g precision) and the biomass data were recorded.

### 4.3. Computational Formula

#### 4.3.1. Importance Value

The importance value (IV) is a comprehensive numerical indicator used to represent the relative importance of a certain species within a community. It is widely applied in vegetation ecology research [48], the calculation formula is as follows:

(1) $$IV=\frac{RH+RA+RAG}{3}$$

(2) $$RH=\frac{H_{i}}{\sum H}$$

(3) $$RA=\frac{A_{i}}{\sum A}$$

(4) $$RAG=\frac{{AGB}_{i}}{\sum AGB}$$

In this formula, RH represents relative height; RA represents relative density; RAG represents relative above-ground biomass. H_i_, A_i_, and AGB_i_ represent the total height, total number, and total above-ground biomass of species i, respectively. ∑H, ∑A, and ∑AGB represent the total height, total number, and total above-ground biomass of all species within the herbaceous sample plot.

#### 4.3.2. Overall Association

The overall association degree of the community was tested based on the variance ratio (VR) proposed by Schluter, and the statistical quantity W was used to evaluate the significance of the connections [49]:

(5) $$VR=\frac{S_{T}^{2}}{\sigma_{T}^{2}}$$

(6) $$\sigma_{T}^{2}=\sum_{i=1}^{S}P_{i}(1-P_{i})$$

(7) $$S_{T}^{2}=(\frac{1}{N})\sum_{i=1}^{N}(T_{j}-t{)}^{2}$$

In this formula, N represents the total number of sample plots, S represents the total number of species, P_i_ represents the frequency of species i, T_j_ represents the total number of species in sample plot j, and t represents the average number of species across all sample plots.

When VR > 1, it indicates that the community as a whole has a positive association; when VR = 1, it indicates that the community as a whole has no association; when VR < 1, it indicates that the community as a whole has a negative association. Additionally, to further verify the significance, W = VR × N is used for testing; if W ≤ χ^2^0.95(N)or W ≥ χ^2^0.05(N), then the overall association is significant (*p* < 0.05); if χ^2^0.95(N) < W < χ^2^0.05(N), then the overall association is not significant (*p* > 0.05).

#### 4.3.3. Interspecific Association

Convert the data of the occurrence of species within the community into a binary data matrix of 0 and 1, establish a 2 × 2 contingency table (as shown in Table 4), and conduct an inter-species association degree test [50,51].

The strength of interspecific associations is analyzed using the connection coefficient AC. The calculation formula is as follows:

When ad ≥ bc, then

(8) $$AC=\frac{\left(ad-bc\right)}{\left(a+b)(b+d\right)}$$

When bc > ad and d ≥ a, then

(9) $$AC=\frac{\left(ad-bc\right)}{\left(a+b)(a+c\right)}$$

When bc > ad and d < a, then

(10) $$AC=\frac{\left(ad-bc\right)}{\left(b+d)(d+c\right)}$$

In this formula, the range of AC is [−1, 1]. The closer AC is to −1, the stronger the negative association between species; the closer AC is to 1, the stronger the positive association between species; when AC is 0, it indicates no association [52]. If AC ≥ 0.6, it indicates a high degree of positive association among various pairs; if 0.3 ≤ AC < 0.6, it indicates a moderate degree of positive association among various pairs; if −0.3 ≤ AC < 0.3, it indicates loose association between species pairs and a tendency towards independence; if −0.6 ≤ AC < −0.3, it indicates a moderate degree of negative association between species pairs; if AC < −0.6, it indicates a high degree of negative association among various pairs.

#### 4.3.4. Interspecific Correlation

The Pearson correlation test assesses the strength of interspecific associations within each shrub understory community, and the calculation formula is as follows:

(11) $$r_{p}(i,j)=\frac{\sum_{k=1}^{n}\left(X_{ik}-{\bar{X}}_{i})(X_{jk}-{\bar{X}}_{j}\right)}{\sqrt{\sum_{k=1}^{n}(X_{ik}-{\bar{X}}_{i}{)}^{2}\sum_{k=1}^{n}(X_{jk}-{\bar{X}}_{j}{)}^{2}}}$$

In the formula, n represents the total number of sample plots; X_ik_ and X_ij_ are the abundance values of species i and j in sample plot k; and is the average abundance value of species i and j across all sample plots. The value range of is [−1, 1]. r_p_(i,j) < 0 indicates negative correlation, r_p_(i,j) = 0 indicates no correlation, and r_p_(i,j) > 0 indicates positive correlation.

#### 4.3.5. Niche Characteristics

Niche width indicates the total range of resources utilized by species within a community, serving as an indicator of the community’s adaptability to environmental conditions. Niche width was calculated based on Levins’ niche widths (B_L_) [53]; The niche overlap index O_ik_ refers to the degree of overlap in the utilization of a certain resource unit by species i and species k. Niche overlap was measured using Pianka’s niche overlap coefficient (O_ik_), the calculation formula is as follows:

(12) $$B_{L}=\frac{1}{\sum_{j=1}^{r}P_{ij}^{2}}$$

(13) $$O_{ik}=\frac{\sum_{j=1}^{r}\left(P_{ij}\times P_{kj}\right)}{\sqrt{\sum_{i=1}^{r}P_{ij}^{2}\times \sum_{j=1}^{r}P_{kj}^{2}}}$$

In the formula, P_ij_ and P_kj_ represent the proportions of the total importance values of species i and k in sample j. r is the total number of samples, B_L_ is the ecological niche width of the species, and O_ik_ is the ecological niche overlap value of the species.

#### 4.3.6. Community Stability

Use the M. Godron stability determination method [54], arrange the plants in the community in descending order of relative frequency, calculate the cumulative percentage of species’ reciprocal values as the independent variable, and calculate the cumulative relative frequency as the dependent variable. Then, a scatter smoothing curve was used to fit the binomial equation, making it intersect with the straight line y = 100 − x. The coordinates of the intersection point represent the desired ratio of community stability. According to the stability judgment criteria proposed by M. Godron, the Euclidean distance between the coordinates of this intersection point and the theoretical stable point (20, 80) is calculated. The smaller the distance value, the higher the stability of the community.

### 4.4. Data Analysis

In this study, the distribution map of the four shrub forest survey plots in the study area was generated using ArcGIS 10.8, and Excel 2021 (Microsoft Corporation, Redmond, WA, USA) was used to initially organize the survey data of plant samples and calculate the species importance values within different communities. The ecological niche characteristic indices (ecological niche width, overlap degree), overall association (variance ratio VR, statistical quantity W), and interspecific association (AC connection coefficient, Pearson correlation coefficient) were calculated using the ‘spaa’ package of R language version 4.2.0 (R Core Team, Vienna, Austria). Other figures such as semi-matrix diagrams and plant community stability analysis were completed in Origin 2024 (OriginLab, Northampton, MA, USA) and R 4.2.0 software.

## 5. Conclusions

Based on a comprehensive analysis of the niche characteristics, interspecific associations, and community stability of understory vegetation in four artificial sand-fixing shrub forests in the Mu Us Sandy Land, this study reveals distinct community assembly mechanisms and restoration statuses among different shrub types. The results showed: (1) Species such as Lc (*Leymus chinensis*), Ee (*Euphorbia esula*), Gd (*Grubovia dasyphylla*), and Ch (*Corispermum hyssopifolium*) function as dominant species with broad niches and high importance values, playing a crucial role in maintaining community function. (2) The understory herbaceous plant communities of *S. psammophila*, *A. ordosica* and *C. fruticosum* exhibited low niche overlap and significant positive overall associations, reflecting clear niche differentiation and relatively stable species coexistence. In contrast, the *A. fruticosa* understory herbaceous plant community displayed high niche overlap, negative overall association, and intense interspecific competition, resulting in the lowest stability. (3) The four plant communities are mainly characterized by no connection and negative correlation, indicating that the spatial distribution of species is highly independent, and the community structure has not yet formed a stable interspecific relationship network. (4) The stability ranking, determined by M. Godron’s method, the stability of herbaceous plant community under *S. psammophila* is the highest, followed by *A. ordosica* and *C. fruticosum*, while the stability of herbaceous plant community under *A. fruticosa* is the lowest, with community stability positively correlated with mean niche overlap and negatively with the variance ratio (VR). *S. psammophila* and *C. fruticosum* have excellent water retention properties and can promote the succession process of plant communities under shrub forests. We recommend prioritizing *S. psammophila* and *C. fruticosum* for sand fixation and conserving key herbaceous species to optimize resource use and stabilize interspecific relationships.

The novelty of this work lies in the integrated assessment of niche characteristics, interspecific associations, and community stability, providing a holistic perspective on the assembly mechanisms of artificial sand-fixing forest ecosystems. However, limitations such as a small sample size and the lack of detailed environmental indicators (soil moisture, nutrients) highlight the need for future research to incorporate environmental factors and shrub functional traits (root architecture, litter properties) to elucidate the synergistic effects of interspecific relationships, shrub growth characteristics, and environmental drivers on community stability, thereby offering a more robust scientific basis for species selection and vegetation restoration management in desert ecosystems.

## Figures and Tables

**Table 1:** Niche width and importance value of understory herbaceous plant community species under different sand-fixing shrubs, including dominant species (DS) and accompanying species (AS).

Community Type	Abbreviation	Latin Name	Species Type	Importance Value (%)	Niche Width
understory herbaceous plant community of *S. psammophila*	Lc	*Leymus chinensis*	DS	16.34	4.07
	Ao	*Artemisia ordosica*	DS	19.18	3.6
	Pt	*Parthenocissus tricuspidata*	DS	14.13	3.27
	Ct	*Cynanchum thesioides*	AS	5.72	2.29
	Ta	*Teloxys aristata*	DS	13.4	2.27
	Ip	*Ixeris polycephala*	AS	2.33	2
	Cs	*Cleistogenes songorica*	AS	1.37	1.8
	Gd	*Grubovia dasyphylla*	AS	8.57	1.6
	Sv	*Setaria viridis*	AS	2.62	1.47
	Aa	*Aster altaicus*	AS	5.3	1
	Ch	*Corispermum hyssopifolium*	AS	2.27	1
	Cc	*Cynanchum chinense*	AS	1.71	1
	Ck	*Cynanchum komarovii*	AS	1.98	1
	Tl	*Thermopsis lanceolata*	AS	1.95	1
	Ac	*Asparagus cochinchinensis*	AS	0.32	1
	Sb	*Stipa breviflora*	AS	2.8	1
understory herbaceous plant community of *A. ordosica*	Ee	*Euphorbia esula*	DS	32.59	3.12
	Pt	*Parthenocissus tricuspidata*	DS	25.98	2.85
	Ed	*Elymus dahuricus*	DS	20.25	2.74
	Sv	*Setaria viridis*	AS	7.82	2.67
	Gd	*Grubovia dasyphylla*	AS	3.92	1.8
	Ta	*Teloxys aristata*	AS	2.78	1.47
	Ct	*Cynanchum thesioides*	AS	0.73	1
	Cs	*Cleistogenes songorica*	AS	4.06	1
	Pte	*Polygala tenuifolia*	AS	0.77	1
	Sb	*Stipa breviflora*	AS	1.09	1
understory herbaceous plant community of *C. fruticosum*	Gd	*Grubovia dasyphylla*	DS	21.17	3.58
	Sv	*Setaria viridis*	AS	6.68	3.52
	Ao	*Artemisia ordosica*	DS	20.32	2.88
	Aa	*Aster altaicus*	DS	14.12	2
	Ch	*Corispermum hyssopifolium*	DS	32.42	1.22
	Cc	*Cynanchum chinense*	AS	2.46	1
	Ip	*Ixeris polycephala*	AS	1.95	1
	Sc	*Silene conoidea*	AS	0.89	1
understory herbaceous plant community of *A. fruticosa*	Ch	*Corispermum hyssopifolium*	DS	50.5	2.27
	Ct	*Cynanchum thesioides*	DS	15.89	2
	Pa	*Phragmites australis*	DS	28.12	1.6
	Cf	*Corethrodendron fruticosum*	AS	5.49	1

**Table 2:** The overall correlation between species of understory herbaceous plant communities under different sand-fixing shrubs.

Community Type	S_T_^2^	σ_T_^2^	Variance Ratio (VR)	df	Test Statistic (W)	χ^2^ Critical Value	*p*	Test Results
understory community of *S. psammophila*	5.44	2.40	2.67	5	11.33	(1.145, 11.071)	0.025	Significant positive association
understory community of *A. ordosica*	4.24	1.76	2.41	5	12.05	(1.145, 11.071)	0.025	Significant positive association
understory community of *C. fruticosum*	2.16	1.28	1.69	5	8.44	(1.145, 11.071)	0.9	Non-significant positive association
understory community of *A. fruticosa*	0.64	0.88	0.73	5	3.64	(1.145, 11.071)	0.9	Non-significant negative association

**Table 3:** Stability of plant community under different sand-fixation shrubs.

Community Type	Polynomial	R^2^	Intersection Coordinates	Test Results	Euclidean Distance
understory community of *S. psammophila*	Y = −0.01X^2^ + 1.93X + 5.62	0.99	37.06, 62.94	Unstable	24.13
understory community of *A. ordosica*	Y = −0.01X^2^ + 1.9X + 3.55	0.99	38.01, 61.99	Unstable	25.47
understory community of *C. fruticosum*	Y = −0.01X^2^ + 2.04X−1.92	0.99	38.27, 61.73	Unstable	25.84
understory community of *A. fruticosa*	Y = −0.01X^2^ + 1.47X + 3.13	0.97	42.85, 57.15	Unstable	32.31

**Table 4:** 2 × 2 contingency table.

Species		Species B		
		Present	Absent	Total
**Species A**	**Present**	a	b	a + b
	**Absent**	c	d	c + d
	**Total**	a + c	b + d	N = a + b + c + d

## Data Availability

Data are contained within the article.
